# The Role of Fibroblast Growth Factor 21 in Diabetic Cardiovascular Complications and Related Epigenetic Mechanisms

**DOI:** 10.3389/fendo.2021.598008

**Published:** 2021-07-19

**Authors:** Mengjie Xiao, Yufeng Tang, Shudong Wang, Jie Wang, Jie Wang, Yuanfang Guo, Jingjing Zhang, Junlian Gu

**Affiliations:** ^1^ School of Nursing and Rehabilitation, Cheeloo College of Medicine, Shandong University, Jinan, China; ^2^ Department of Orthopedic Surgery, The First Affiliated Hospital of Shandong First Medical University, Jinan, China; ^3^ Department of Cardiology at the First Hospital of Jilin University, Changchun, China; ^4^ Department of Cardiology at the First Hospital of China Medical University, and Department of Cardiology at the People’s Hospital of Liaoning Province, Shenyang, China

**Keywords:** FGF21, epigenetic, diabetes, cardiovascular complications, mechanisms

## Abstract

Fibroblast growth factor 21 (FGF21), is an emerging metabolic regulator mediates multiple beneficial effects in the treatment of metabolic disorders and related complications. Recent studies showed that FGF21 acts as an important inhibitor in the onset and progression of cardiovascular complications of diabetes mellitus (DM). Furthermore, evidences discussed so far demonstrate that epigenetic modifications exert a crucial role in the initiation and development of DM-related cardiovascular complications. Thus, epigenetic modifications may involve in the function of FGF21 on DM-induced cardiovascular complications. Therefore, this review mainly interprets and delineates the recent advances of role of FGF21 in DM cardiovascular complications. Then, the possible changes of epigenetics related to the role of FGF21 on DM-induced cardiovascular complications are discussed. Thus, this article not only implies deeper understanding of the pathological mechanism of DM-related cardiovascular complications, but also provides the possible novel therapeutic strategy for DM-induced cardiovascular complications by targeting FGF21 and related epigenetic mechanism.

## Introduction

Fibroblast growth factor 21 (FGF21), belongs to the FGF family, is mainly expressed in the liver, adipose tissues and skeletal muscle (1, 2). FGF21 action is mediated by FGF receptors (FGFRs) and β-klotho (a single-pass transmembrane protein, known as a co-receptor for cellular responsiveness to FGF21 action) (1, 2). Endogenous FGF21 has been proposed to be a hormone to maintain lipid and glucose metabolism under both physiological and pathological conditions (2–6). In addition, FGF21 also plays a critical role in the treatment of cardiovascular diseases (7, 8). For example, high serum level of endogenous FGF21 is considered as a compensatory response to ameliorate atherosclerotic diseases or represents the resistant state of FGF21 (7, 9), while treatment with exogenous FGF21 could protect against atherosclerosis (10). Particularly, several lines of evidences indicate a close and complicated relationship between DM-induced cardiovascular complications and FGF21 (11–13). Studies have shown that the early compensatory serum high level of FGF21 is responsive to the occurrence and development of DM-induced cardiovascular complications (14, 15). While the deletion of FGF21 could aggravate the DM-induced cardiovascular injury (16, 17). Furthermore, exogenous FGF21 has been shown could improve DM-induced cardiovascular injury in rodents (11, 18). The cardiovascular protective effect of exogenous FGF21 is mainly mediated by the anti-oxidative stress (11), anti-inflammatory (19), anti-apoptosis (20) and lipid-lowering effects (21). However, despite as a biomarker and diagnostic indicator of DM-related cardiovascular diseases in clinic (14, 22), the clinical implementation of FGF21 still has some obstacles due to its complex pharmacokinetic and biophysical characteristics (23).

To date, accumulating evidences have demonstrated that hyperglycemia could result in continuous cardiovascular complications despite achievement of glycemic control, which is called “metabolic memory” (24–26). Metabolic memory is related to the epigenetic modifications without the change of DNA sequence, including modifications of chromatin histone, methylation of DNA, and gene regulations by non-coding RNAs (25). Thus, a deep study of the epigenetic modifications and formulation of corresponding treatment strategies are beneficial to the prevention and treatment of diabetic cardiovascular complications. Moreover, the role of epigenetic mechanism related to FGF21 in the treatment of DM and associated complications has attracted extensive attention of researchers. For example, it is reported that the inhibition of histone deacetylase 3 (HDAC3) could up-regulate *Fgf21* gene transcription to ameliorate DM-induced vascular injury (27). It has also been shown that exogenous FGF21 treatment might increase microRNA (miRNA)-155-3p and miRNA-1968-5p to control hepatic energy metabolism in the state of insulin resistance (28). In addition, according to a recent study, administration of FGF21 in an obese mice model could improve hepatic steatosis and autophagy through upregulating autophagy genes *via* demethylation of lysine 27 on histone 3 (H3K27) (29). Therefore, the purpose of this review is to analyze the effects and related mechanisms of FGF21 on DM-related cardiovascular complications. Moreover, the possible epigenetic changes that may be related to the function of FGF21 on DM-associated cardiovascular complications is discussed, so as to provide reference for further studies.

## FGF21 and Diabetic Cardiomyopathy

**Figure 1:**
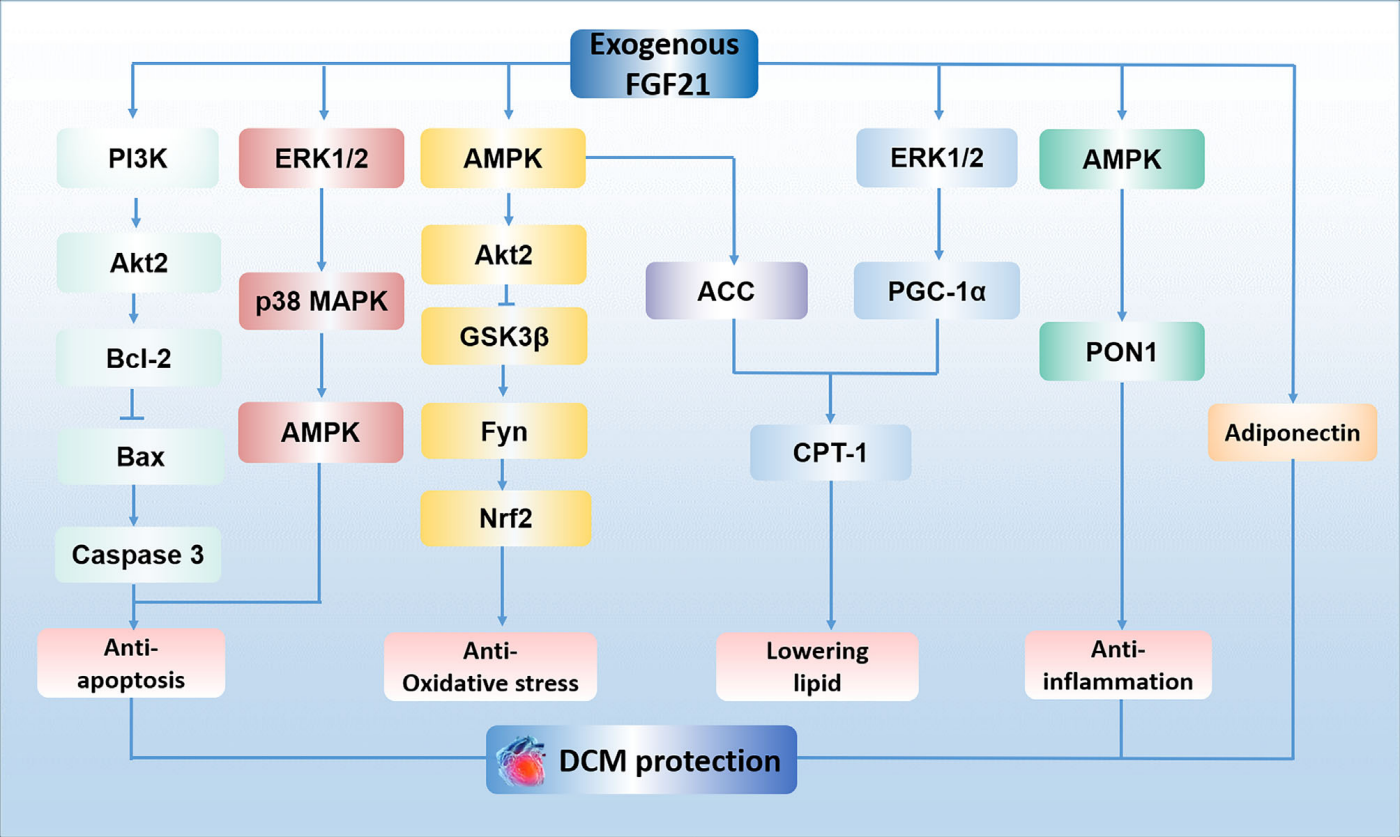
The mechanisms of exogenous FGF21 action in DCM treatment. FGF21 plays an anti-apoptosis role in cardiomyocytes by activating PI3K/Akt2/Bcl-2/Bax/Caspase 3 pathway and ERK1/2/p38 MAPK/AMPK pathway. FGF21 protects against oxidative stress by activating AMPK/Akt2/GSK3β/Fyn/Nrf2 pathway. FGF21 lowers lipid accumulation *via* activating AMPK/ACC/CPT-1 pathway and ERK1/2/PGC-1α/CPT-1 pathway in the myocardium. FGF21 reduces inflammation in cardiomyocytes by upregulating AMPK/PON1 signaling. The effect of FGF21 on the treatment of DCM is also regulated by adiponectin. PI3K, phosphatidylinositol-3-kinase; Akt2, protein kinase B; Bcl-2, B-cell lymphoma-2; Bax, Bcl-2-associated X protein; ERK1/2, extracellular signal-regulated kinase 1/2; p38 MAPK, mitogen-activated protein kinase 14; AMPK, adenosine 5′-monophosphate (AMP)-activated protein kinase; GSK3β, glycogen synthase kinase-3β; Nrf2, nuclear factor (erythroid-derived 2)-like 2; ACC, acetyl-CoA carboxylase; CPT-1, carnitine palmitoyltransferase 1; PGC-1α, peroxisome proliferator-activated receptor-γ coactivator-1α; PON1, paraoxonase 1; DCM, diabetic cardiomyopathy.

Diabetic cardiomyopathy (DCM) is defined as a chronic myocardial disorder caused by DM, and its onset is not related to hypertension, coronary artery disease, and valvular heart disease (30, 31). Hyper-glycaemia (32), insulin resistance (32), micro-vascular lesions (33) and calcium overload in cardiomyocytes (31) were reported to be involved in this disorder. Mechanisms such as oxidative stress (34), lipid metabolism imbalance (35), inflammatory response (36), autophagy suppression (37), as well as myocardial cell apoptosis (37) are key factors to facilitate the progression of DCM. Recently, a growing body of evidence demonstrates that FGF21 may be an effective drug for the treatment of DCM, especially in the aspects of reducing oxidative stress (19), inflammatory (19), apoptosis (20) and lowering lipid (21) in the myocardium. For instance, Wu et al. demonstrated FGF21 reduces inflammation in cardiomyocytes by upregulating adenosine 5′-monophosphate (AMP)-activated protein kinase (AMPK)/paraoxonase 1 (PON1) signaling (19). Zhang et al. have shown that FGF21 alleviates DM-related cardiac apoptosis *via* activating the extracellular signal-regulated kinase 1/2 (ERK1/2)/mitogen-activated protein kinase 14 (p38 MAPK)/AMPK pathway in a mice model of type 1 DM (T1DM) (20). Besides, a previous study demonstrated that FGF21 also exerts lipid-lowering and anti-oxidative effect through activating AMPK/acetyl-CoA carboxylase (ACC)/carnitine palmitoyltransferase-1 (CPT-1) pathway and AMPK/protein kinase B (Akt2)/glycogen synthase kinase-3β (GSK3β)/Fyn/nuclear factor (erythroid-derived 2)-like 2 (Nrf2) pathway in a mice model of type 2 DM (T2DM) (21). Furthermore, long-term treatment of FGF21 could improve cardiac mitochondrial redox homoeostasis and structural changes by activating ERK1/2/peroxisome proliferator-activated receptor-γ coactivator-1α (PGC-1α)/CPT-1 lipid-lowering pathway and phosphatidylinositol-3-kinase (PI3K)/Akt2/B-cell lymphoma-2 (Bcl-2)/Bcl-2-associated X protein (Bax)/Caspase 3 anti-apoptotic pathways in an obese, insulin-resistant rat model with FGF21 resistance (18). In addition, it is reported that administration of FGF21 could increase serum level of adiponectin (a kind of hormone has been proved to exert cardioprotective effect (38)), suggesting the cardioprotective effect of FGF21 on DCM may be regulated by increasing adiponectin level in serum (18). However, the existence of FGFR1 and β-klotho in the myocardium indicates that FGF21 may also directly protect the heart against DM (39, 40). Indeed, previous study proved FGF21 strongly improved high-glucose (HG)-induced oxidative stress and fibrosis in primary mouse cardiomyocytes, and these protective effects of FGF21 were markedly weakened by genetic blockage of β-klotho (19), suggesting FGF21 ameliorates DCM may be mediated by its direct action on the heart. Therefore, FGF21 is considered as a promising candidate for the therapy of DCM. The mechanisms of exogenous FGF21 action in DCM treatment is presented in **Figure 1**.

## Epigenetic Regulation Related to FGF21 in Diabetic Cardiac Complications

### Histone Modifications

Epigenetic changes of histones, such as methylation, phosphorylation, acetylation, and ubiquitination, are key factors contribute to the development of chronic diabetic complications (25). Histone deacetylases (HDACs) are one of the vital cellular regulators that could regulate histone deacetylation (41). Xu et al. reported that activity of HDAC3 is significantly enhanced in the heart of diabetic mice. They further found that HDAC3 inhibition suppresses DM-induced oxidative stress and inflammation to improve cardiac dysfunction and remodeling in the diabetic mice (42). Actually, endogenous FGF21 could also be regulated by HDAC3 (43–45). Remarkably, a previous study has demonstrated that HDAC3 inhibition could result in FGF21 secretion and then lead to the reduction of aortic fibrosis and inflammation in a diabetic mice model, mechanistically, inhibition of HDAC3 may promote Nrf2 activity by the up-regulation of miRNA-200a expression with a down-regulation of kelch-like ECH-associated protein 1 (Keap1) to preserve expression of hepatic FGF21 (27). However, whether attenuation of DCM is regulated by FGF21/HDAC3 requires further exploration.

### Non-Coding RNAs

Except for histone modifications, non-coding RNAs (ncRNAs) also have been implicated as new participants in the pathogenesis of DM-associated complications. In general, ncRNAs, including miRNAs, circular RNAs (cirRNAs), and long non-coding RNAs (lncRNAs) (46), act as important regulators in controlling gene transcription and protein expression. Of note, miRNAs could base pair with specific target mRNAs to control expression of gene and regulate a series of biological functions (47–49). The aberrantly expression of miRNAs in human diseases showing the therapeutic potential by targeting miRNAs (25). Recently, Costantino et al. showed that the up-regulation of miRNA-34 and miRNA-218 induced by hyperglycaemia in the heart leads to persistent oxidative stress, while inhibition of these miRNAs reduces oxidative stress and restores left ventricular dysfunction (50). Furthermore, it is reported that obesity-induced elevated miRNA-34a could suppress sirtuin 1 (SIRT1) function and adipocyte FGF21, while downregulation of miRNA-34a could improve hepatic FGF21 signaling to alleviate adiposity (51). In addition, Zhang et al. reported that activated FGF21/SIRT1 pathway by fenofibrate could increase cardiac autophagy to improve fibrosis and inflammation induced by DM (52). Thus, it is possible that downregulation of miRNA-34a may increase the activation of FGF21/SIRT1 to improve DCM. In terms of FGF21 regulating miRNAs, Li et al. reported that FGF21 improves ischemic arrhythmias *via* inhibiting miRNA-143/early growth response protein 1 pathway in a myocardial infarction model (53), yet, the effect of FGF21/miRNA-143 in regulating DCM needs further investigation.

### DNA Methylation

DNA methylation always leads to gene silencing, which mainly occurs on the cytosine ring of ‘CpG islands’ in the 5′ regulatory regions of genes. Besides, DNA methylation is regulated by DNA methyltransferases (Dnmts) (54). You et al. have shown that *Fgf21* gene is a key target of Dnmt3a in the occurrence of adipose insulin resistance. Expression of endogenous FGF21 is decreased by Dnmt3a-mediated DNA methylation at the *Fgf21* promoter, while exogenous FGF21 can restore Dnmt3a overexpression-induced insulin resistance (55). A previous report illustrated that DNA demethylation of *Fgf21* gene induced by peroxisome proliferator activate receptor α (PPARα) during the postnatal period could increase hepatic FGF21 expression, which may partly attenuate diet-induced obesity in adulthood (56). However, the involvement of Dnmt3a and *Fgf21* gene methylation in diabetic cardiac complications is not fully studied.

A recent evidence demonstrated that DM-induced cardiac fibrosis and remodeling could be ameliorated by Salvianolic acid B *via* enhancing DNA methylation on the promoter of *insulin-like growth factor-binding protein 3* (*IGFBP3*). Mechanistically, the suppression of IGFBP3 could increase the phosphorylation of ERK and AKT activities, which ultimately lead to the improvement of left ventricular dysfunction of diabetic mice (57). As we have mentioned that FGF21 could improve DCM by the activation of ERK (20) and AKT (21) pathway, thus, whether FGF21 could ameliorate DCM through inducing DNA methylation on *IGFBP3* requires further investigation.

In conclusion, all these findings suggest that expression of endogenous FGF21 is closely related to epigenetic modification in multiple pathological conditions. However, lots of work remain to be done to fully understand the relationship between epigenetic regulation and FGF21 in DM-induced cardiac complications.

## FGF21 and Diabetic Vascular Complications

### FGF21 and Endothelial Dysfunction Induced by DM

The HG environment could reduce the ratio of Ser^1177^/Thr^495^ phosphorylation of endothelial nitric oxide synthase (eNOS) and increase inflammatory response to destroy endothelial function in diabetic mice (58). This dysfunction of endothelial cells (ECs) is not only considered as a well-accepted marker, but also a starting point for angiopathy in DM (59). Endogenous FGF21 and β-klotho could be upregulated by HG, while administration of FGF21 could prevent HG-induced cellular damage and eNOS dysfunction in ECs (60). Moreover, previous study demonstrated that exogenous FGF21 inhibits oxidative stress and apoptosis induced by HG in ECs through the activation of PI3K/Akt/forkhead-box type O 3a signaling pathway (61). Ying et al. suggested FGF21 could directly suppress oxidative stress and enhance endothelium-dependent vasorelaxation of aorta through the activation of calcium/calmodulin-dependent protein kinase kinase 2/AMPKα pathway in both T1DM and T2DM mice model, and that is independent of its glucose-lowering and insulin-sensitizing effects. Additionally, the FGFR and β-klotho has been proved to be expressed in ECs, and accordingly the protective effect of FGF21 on inhibiting oxidative stress could be blocked by FGFR antagonist (11). These above findings suggest that FGF21 could be a promising therapeutic drug for vascular complications induced by DM.

### FGF21 and Atherosclerosis Induced by DM

Atherosclerosis, a chronic and progressive disease in the large-sized arteries, is characterized by the accumulation of lipids in the artery (62). Compared with non-DM patients, DM significantly enhances brachial–ankle pulse wave velocity, increases vascular intima-medial thickness, and formats a mass of plaques in the artery. These pathologic changes are postulated to be mechanisms for the subclinical atherosclerosis (initiation stage of atherosclerosis) (63). It is reported that elevated serum level of FGF21 was related to atherosclerosis in subjects with DM (12, 14, 15, 64), suggesting FGF21 resistance and/or a compensatory mechanism in response to DM. Yan et al. found that deletion of FGF21 in diabetic mice could worsen DM-induced cell apoptosis and aortic remodeling by aggravating aortic oxidative stress and inflammation, while the pathologic changes caused by FGF21 knockout was reversed by exogenous FGF21 administration in diabetic mice (17). Recently, Kim et al. also demonstrated that FGF21 combined with glucagon-like peptide-1 analogue could strongly ameliorate atherosclerosis-related process induced by T2DM in a mice model (65). Therefore, the above results indicate that FGF21 may act as not only a biomarker but also a promising therapeutic agent for atherosclerosis induced by DM.

Moreover, neointima hyperplasia, as the pathological base of atherosclerosis, has been found to be related to DM. Wei et al. have shown FGF21 could significantly prohibit neointima hyperplasia possibly through the inhibition of spleen tyrosine kinase (Syk)/leucine-rich repeat (LRR)-containing protein 3 (NLRP3) inflammasome pathway in diabetic mice (13). Given that FGFR1 is highly-expressed in aorta (66), Wei et al. further found that the inhibiting effect of FGF21 on Syk and NLRP3 inflammasome activity is abolished by FGFR1 inhibitor in vascular smooth muscle cells (VSMCs) (13), which indicates that FGF21 may directly act on VSMCs to improve atherosclerosis.

Perivascular adipose tissue (PVAT), a vessel-supporting connective tissue (67), could protect against blood vessels injury induced by DM (68). Chang et al. found that the activation of PVAT could attenuate atherosclerosis (69). Berti et al. demonstrated that exogenous FGF21 may protect against atherosclerosis by inducing PVAT to release omentin 1 (70). Herein, FGF21-medieted PVAT activation is indicated to be an effective method for the treatment of DM-related atherosclerosis. Expression of endogenous FGF21 and its possible function in different diabetic atherosclerosis is described in **Table 1**.

**Table 1:** Expression of endogenous FGF21 and possible function in different diabetic atherosclerosis.

Type of disease	Type of artery	Expression of FGF21	Possible function	Reference
Subclinical atherosclerosis and arterial stiffness in T2DM	Carotid artery	Up	A biomarker of subclinical atherosclerosis and arterial stiffness	(12)
Subclinical atherosclerosis in T2DM	Carotid, iliac artery	Up	A compensatory reaction to offset atherosclerosis	(14)
Carotid artery plaque in T2DM	Carotid artery	Up	A compensatory mechanism and/or FGF21 resistance	(15)
LEAD in T2DM	Lower extremity artery	Up	An independent risk factor for LEAD in type 2 diabetic women	(64)

LEAD, lower extremity atherosclerotic disease; T2DM, type 2 diabetes mellitus.

### FGF21 and Vascular Calcification Induced by DM

Vascular calcification (VC) is considered as an important complication induced by DM (71). The main pathological changes in VC including the decreased compliance of the vascular wall and increased stiffness, which easily lead to a multiple of adverse cardiovascular events (72). It is reported that DM may promote VC by enhancing the expression of inflammatory cytokines, activating bone morphogenetic proteins pathway and receptor activator of nuclear factor-kβ (RANK)/RANK ligand pathway (73). Recently, Gan et al. demonstrated that lower baseline level of FGF21 in serum could predict a better long-term prognosis in patients with both DM and coronary artery calcification (22). But it has been proved that the application of FGF21 could resist to the calcification of VSMCs by the activation of FGFR1/3/β-klotho/P38/MAPK/runt-related transcription factor 2 signaling pathway (72). Furthermore, Shi et al. indicated that exogenous FGF21 could perform an anti-calcifying effect by inhibiting endoplasmic reticulum stress-induced apoptosis in a rat model (74). However, the anti-calcifying role of FGF21 in the treatment of DM-induced vascular calcification and its related pathway needs further investigation.

## Epigenetic Regulation Related to FGF21 in Diabetic Vascular Complications

### Histone Modifications

In recent years, emerging evidences have shown that histone modifications are related to vascular dysfunction triggered by DM (75, 76). Several evidences demonstrated that histone methylation of the *Fgf21* promoter may involve in the development of diabetic vascular complications. Claycombe et al. showed that expression of histone methyltransferase G9a is increased and transcription of *Fgf21* gene is decreased in an obesity and insulin resistance rat model (77). Also, it is reported that the transcriptional repression of *Fgf21* could be mediated by histone methyltransferase G9a through increasing dimethylation at lysine 9 on histone 3 (H3K9-me2) of the *Fgf21* promoter during refeeding (78). Based on the above studies, it appears that down-regulation of histone methylation of the *Fgf21* promoter by decreasing the expression of histone methyltransferase G9a may be a potential therapeutic strategy to improve vascular complications related to DM.

HDAC inhibition has been found to be successful for improving diabetic vascular complications (27, 76). Besides, several pieces of evidences converge to suggest that the inhibition of HDAC may increase the transcription of *Fgf21* gene to protect against vascular complications induced by DM. Our previous study has shown that HDAC3 inhibition may promote hepatic FGF21 synthesis and elevate serum protein level of FGF21, which contribute to improve DM-induced aortic inflammation and associated pathologies (27). Moreover, it is reported that NaB could increase *Fgf21* gene transcription by inhibiting the activity of HDAC3 to improve fatty acid oxidation and stimulate ketone body production in a dietary obese mice model (45). In addition, sodium butyrate (NaB), has been shown to attenuate aortic endothelial dysfunction induced by DM *via* inhibiting HDAC3 activity (76). Also, grape seed procyanidin extract, as a strong inducer of *Fgf21*, could indirectly increase expression of *Fgf21* gene and protein by the inhibition of HDAC and subsequent activation of PPARα, thereby, exert therapeutic effect on hypertriglyceridemia (79). All these findings indicate that blocking the activity of HDAC could increase *Fgf21* gene expression, which may lead to a recovery in DM-induced vascular complications.

Moreover, SIRT1, belongs to HDACs, has also been found to improve hyperglycemia-induced endothelial dysfunction by deacetylating histone 3 (H3) at the *p66^Shc^* promoter (80). It is well-documented that the protective effect of exogenous FGF21 on obesity and T2DM is closely depends on the activation of SIRT1 and subsequently leads to the deacetylation of its downstream targets, PGC-1α and H3 in human adipocytes (81). Of note, previous study has demonstrated that the protective effect of exogenous FGF21 on diabetic heart is strongly correlated with SIRT1 activity, and the increasement of FGF21 may promote SIRT1-mediated autophagy to prevent pathological and functional abnormalities of heart induced by T1DM (52). These findings implicated that SIRT1 may act as an important factor in mediating the protective effect of exogenous FGF21 on the DM-related vascular complications, however, the related specific epigenetic mechanisms need to be further explored in the future.

### Non-Coding RNAs

Up to now, the role of ncRNAs in DM-mediated vascular injury has been widely explored. lncRNAs, a kind of ncRNAs that more than 200 nucleotides in length (82), have been identified as crucial epigenetic regulators in a variety of biological processes, including act as molecular sponges or scaffolds for certain molecules (83). Zhang et al. have shown that lncRNAs participate in the treatment of diabetic vascular complication. They demonstrated that the overexpression of lncRNA MEG3 may inhibit the expression of TGF-β1 and VEGF to ameliorate diabetic retinopathy, which suggests the up-regulation of the lncR MEG3 as a promising therapy for diabetic vascular complications (84). However, Wan et al. reported that overexpression of lncR AK005401 exacerbates hippocampal injury induced by acute ischemia/reperfusion through significantly increasing the expressions of Yin Yang 1 and decreasing expression of FGF21 to result in reactive oxygen species (ROS) generation, cell apoptosis and mitochondria injury (85). Whether endogenous FGF21 could be inhibited by lncRNAs AK005401 in the diabetic vascular complications needs to be further explored.

The elevated expression of miRNA-34a could lead to diabetic endothelial dysfunction by downregulation of SIRT1 in diabetic mice (86). Also, it is reported that inhibition of miRNA-34a is able to prevent HG-mediated impaired angiogenesis in mouse microvascular ECs by increasing the expression of SIRT1 (87). As we have known that the upregulation of miRNA-34a in obesity restrains fat browning partly by the suppression of FGF21 signaling and SIRT1, while down-regulation of miRNA-34a could upregulate the expression of FGFR1, β-klotho and SIRT1 function to reduce adiposity (51), thus it is convincible that miRNA-34a inhibition may attenuate diabetic vascular complications by improving hepatic FGF21 signaling.

It has been found that endogenous FGF21 expression is downregulated, but sterol regulatory element-binding protein 2 (SREBP2) expression is upregulated in a rat experimental atherosclerosis model (88). Xue et al. reported that FGF21 and glucagon-like polypeptide 1 improve lipid metabolism in diabetic mice by downregulating the expression of the SREBP1/2 genes (89). Moreover, Lin et al. has shown that replenishment of FGF21 could reduce cholesterol synthesis and attenuate hypercholesterolemia in apolipoprotein E^–/–^ mice by the inhibition of SREBP2 hepatic expression (10). The above results indicate that FGF21 could prevent atherosclerosis *via* downregulating expression of SREBP2. Moreover, it is reported that miRNA-33 could interact with SREBPs to aggravate atherosclerosis by affecting macrophage actions (90). Therefore, it is believed that FGF21 prevents atherosclerosis may be mediated by inhibiting the expression of miRNA-33 to repress SREBP2 hepatic expression (91). In addition, although no directly evidence showed that miRNA-33 was involved in the treatment of DM-induced vascular dysfunction so far. Yang et al. has shown that miRNA-33 acts as a key regulator in gestational DM of pregnancy (92). Thus, it is still worth for further exploration on the role of miRNA-33 in the effect of FGF21 on DM-induced atherosclerosis.

Under normal conditions, cirRNAs are key regulators of multiple biological processes by being translated themselves or by regulating protein function, by acting as microRNA or protein inhibitors (93). Moreover, cirRNAs are crucial regulators in the pathogenesis of many metabolic diseases. Of note, it is reported cirRNAs are related to the regulation of β-cell activity in the development of DM (94). In addition, microarray profiling of cirRNA revealed a total of 95 differentially expressed cirRNAs in human ECs under hyperglycaemic conditions, which confirmed the key regulatory role of cirRNAs in DM (95). However, whether cirRNAs involve in the protective effect of FGF21 on diabetic vascular complications is still unknown.

### DNA Methylation

**Figure 2:**
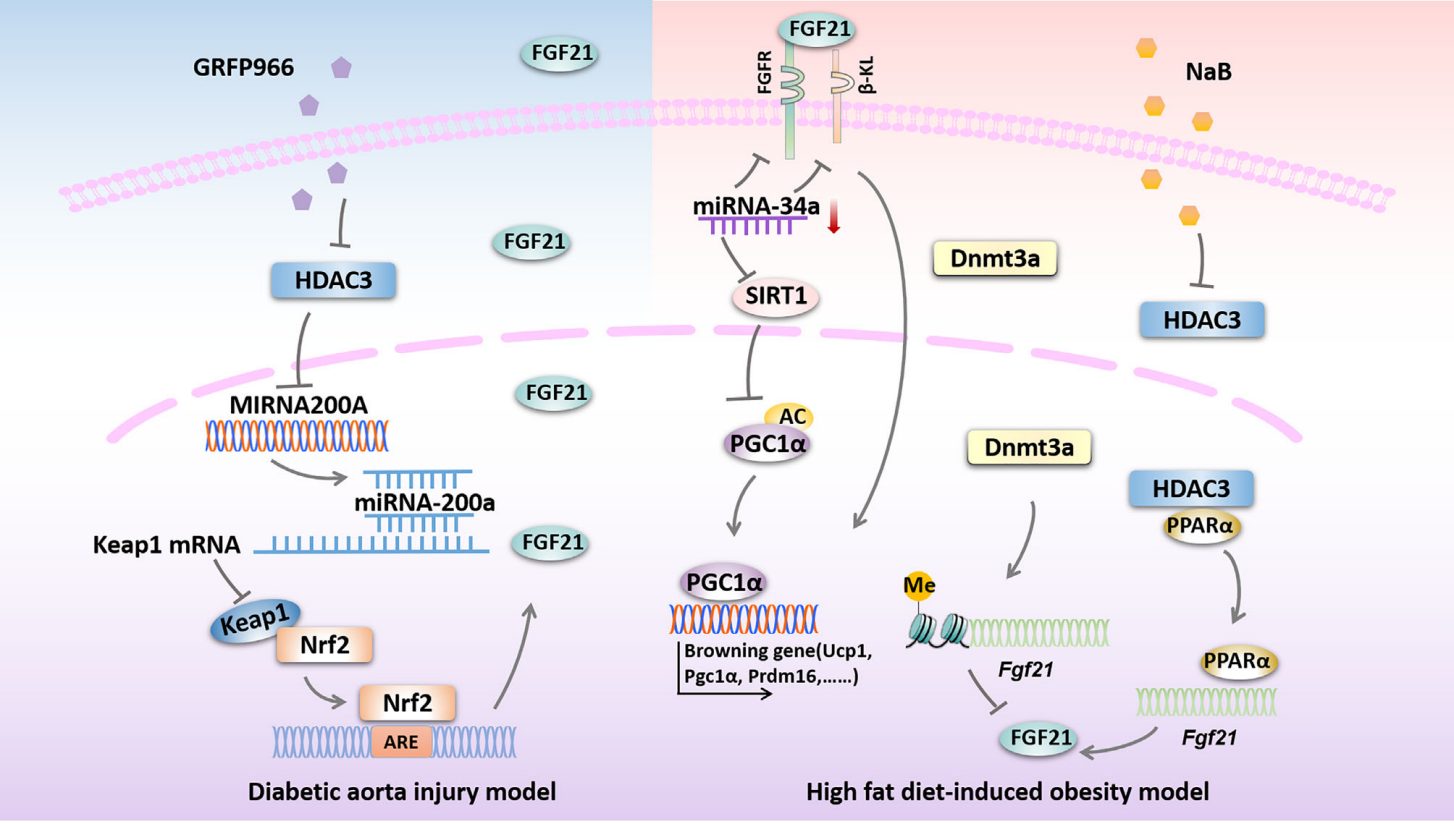
Possible epigenetic modifications targeting FGF21 in diseases related to DM. HDAC3 inhibition by GRFP966 promotes Nrf2 activity *via the* up-regulation of miRNA-200a expression with down-regulation of keap1 to preserve expression of hepatic FGF21 in diabetic aorta injury model. In high fat diet-induced obesity model, down-regulation of miRNA-34a improves hepatic expression of FGF21 signaling and SIRT1 to promote browning fat formation. Expression of FGF21 is reduced by Dnmt3a-mediated DNA methylation at the *Fgf21* promoter. NaB increases *Fgf21* gene transcription by inhibiting HDAC3 interacts with PPARα in the *Fgf21* promoter. HDAC3, histone deacetylase 3; miRNA-200a, microRNA-200a; Keap1, kelch-like ECH-associated protein 1; Nrf2, Nuclear factor (erythroid-derived 2)-like 2; ARE, AU-rich element; FGFR, fibroblast growth factor receptor; β-KL, β-klotho; miRNA-34a, microRNA-34a; SIRT1, sirtuin 1; PGC1α, peroxisome proliferator-activated receptorγcoactivator-1 α; Ucp1, uncoupled protein 1; Prdm16, PRD1-BF1 and RIZ1 homeodomain protein 16; Dnmt3a, DNA methyltransferase 3a; NaB, sodium butyrate; PPARα, peroxisome proliferator activate receptor α.

A couple of studies proposed that DNA methylation is closely related to the development of diabetic vascular complications (96, 97), while the role of *Fgf21* methylation in diabetic vascular complications remains largely unknown. It has been shown that DNA methylation at the *Fgf21* locus was increased in human DM subjects, which is mediated by Dnmt3a and ultimately lead to insulin resistance (55). Moreover, Yuan et al. demonstrated that the PPARα-dependent *Fgf21* demethylation occurs in the liver during the postnatal period, also, they propose that *Fgf21* methylation, as a form of epigenetic memory, could persist into adulthood and play a key role in the developmental progress of obesity (56). These above studies suggest that targeting endogenous *Fgf21* gene methylation could also be a potential method for the treatment of DM and related vascular complications. However, whether exogenous FGF21 could ameliorate DM-induced vascular complications by altering the DNA methylation patterns of specific genes remains to be further studied (54). Possible epigenetic modifications targeting FGF21 in diseases related to DM are presented in **Figure 2**.

## Conclusion

Existing evidences have proved the strong protective effects of FGF21 on diabetic cardiovascular complications, such as inhibition of fibrosis and anti-oxidative stress, as well as reduction of apoptosis and inflammation levels in different diabetic cardiovascular complications models. Remarkably, a body of evidence indicating that epigenetic changes are closely involved in FGF21 and cardiovascular complications of DM, including modification of histone, ncRNAs and DNA methylation, which often occur simultaneously and work together in cardiovascular complications of DM. However, related mechanisms remain not fully elucidated. In particular, the discussion about the relationship between DNA methylation and FGF21 in the diabetic cardiovascular complications still in an early stage. Therefore, deeper research must to be carried out in the future.

## Author Contributions 

MX, JW (4th author), JW (5th author), and YG performed the systematic search, did data extraction, interpreted the data and drafted the review. YT, SW, and JZ contributed to the discussion. JG supervised and revised the manuscript. All authors contributed to the article and approved the submitted version.

## Funding

This study was supported by Qilu Young Scholar’s Program of Shandong University (21330089963007), National Natural Science Foundation of China (81770375) and Jilin Science and Technology Department (20200801061GH).

## Conflict of Interest

The authors declare that the research was conducted in the absence of any commercial or financial relationships that could be construed as a potential conflict of interest.
